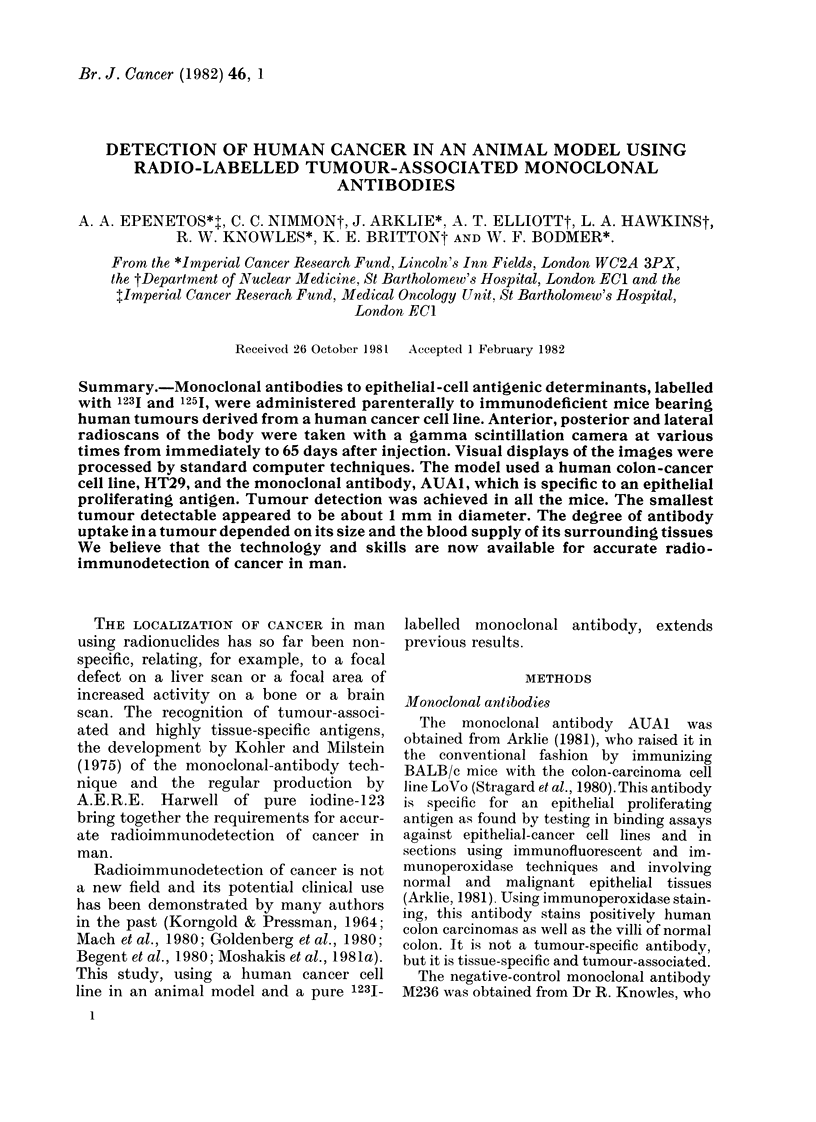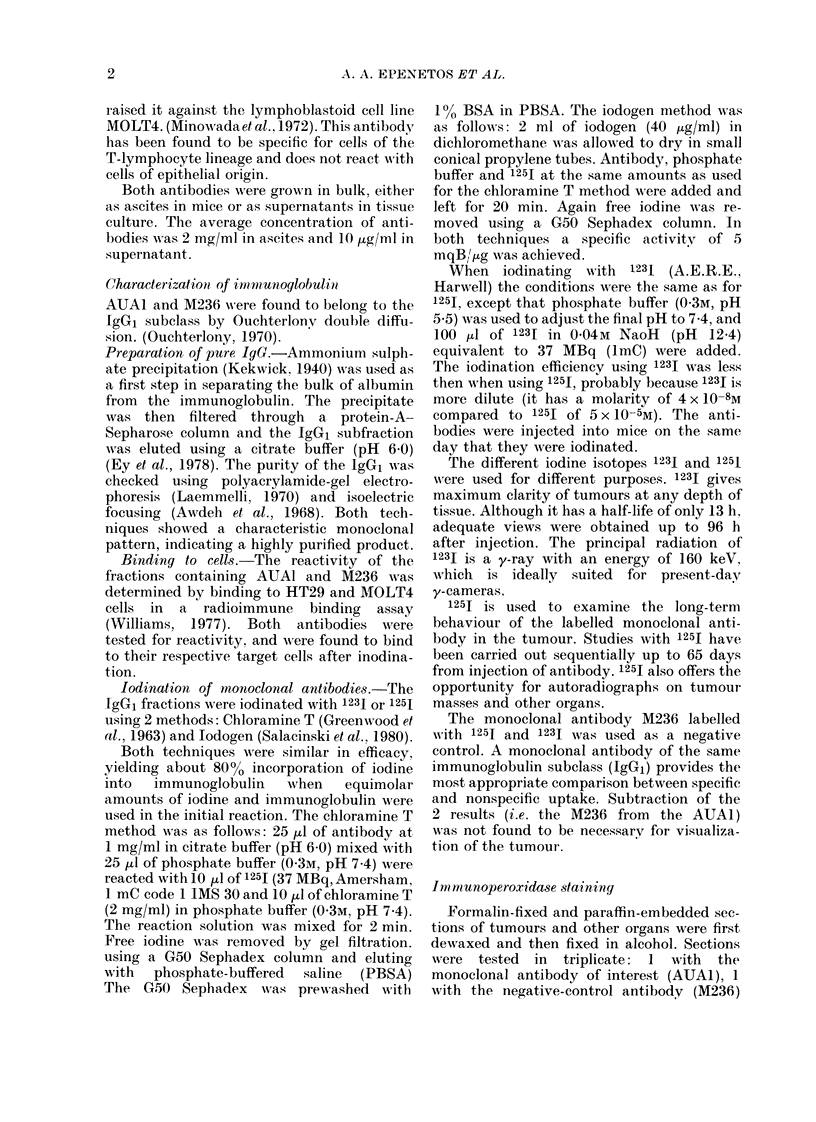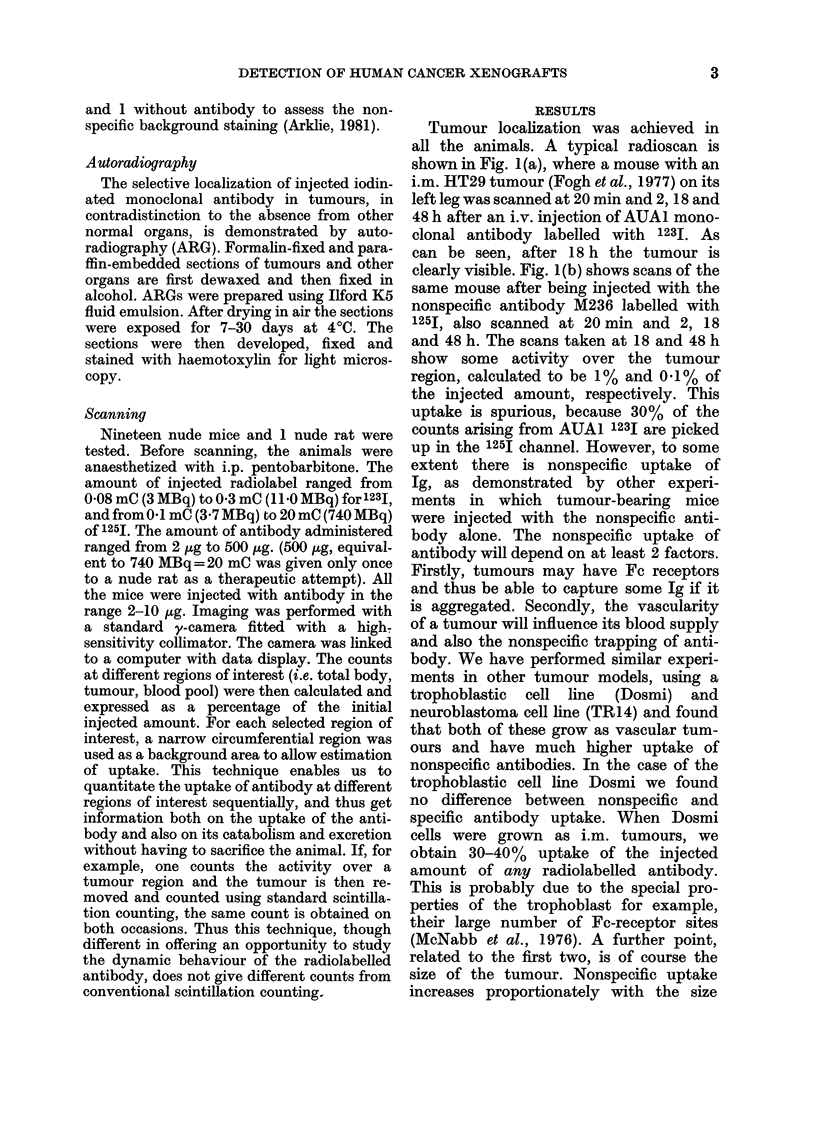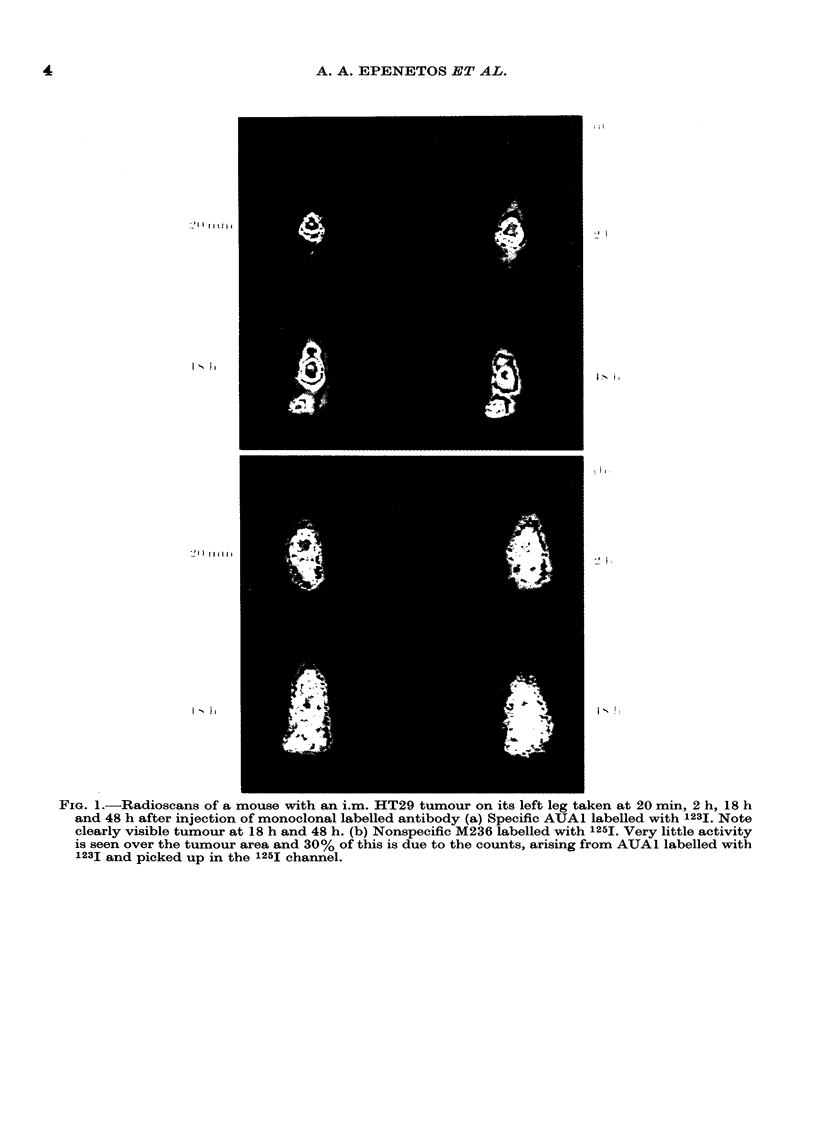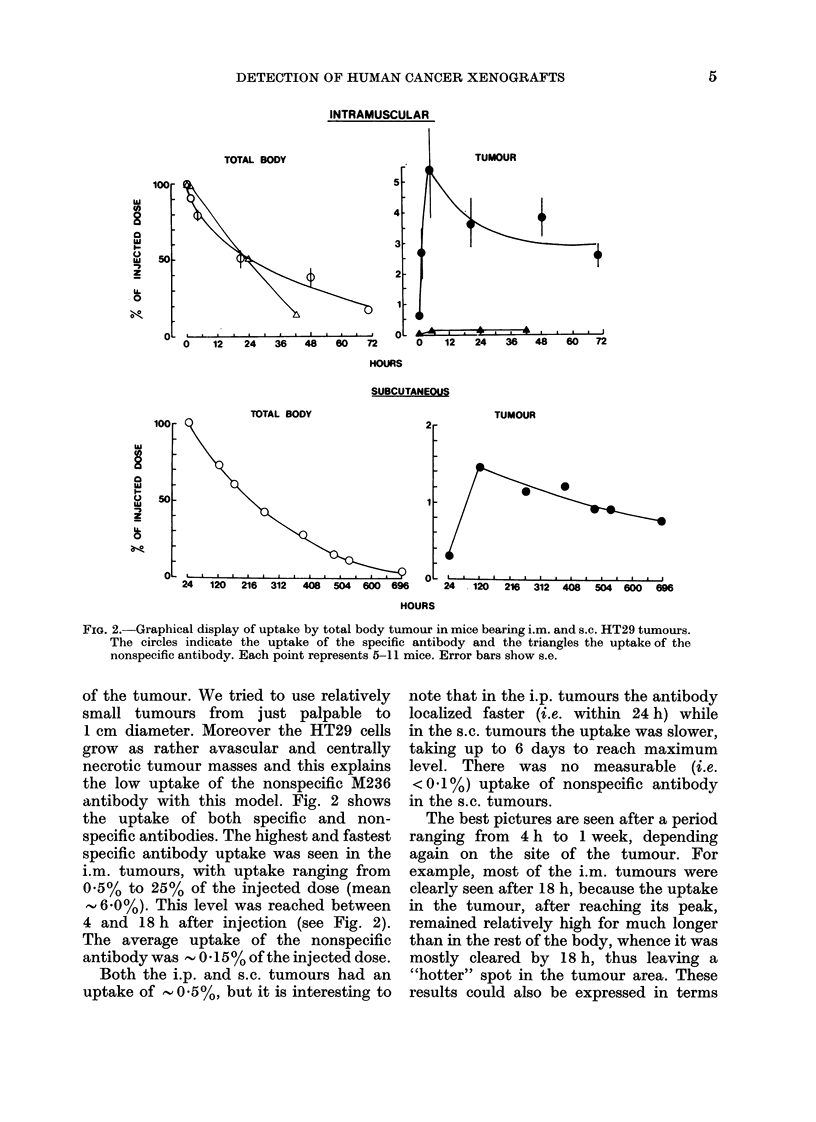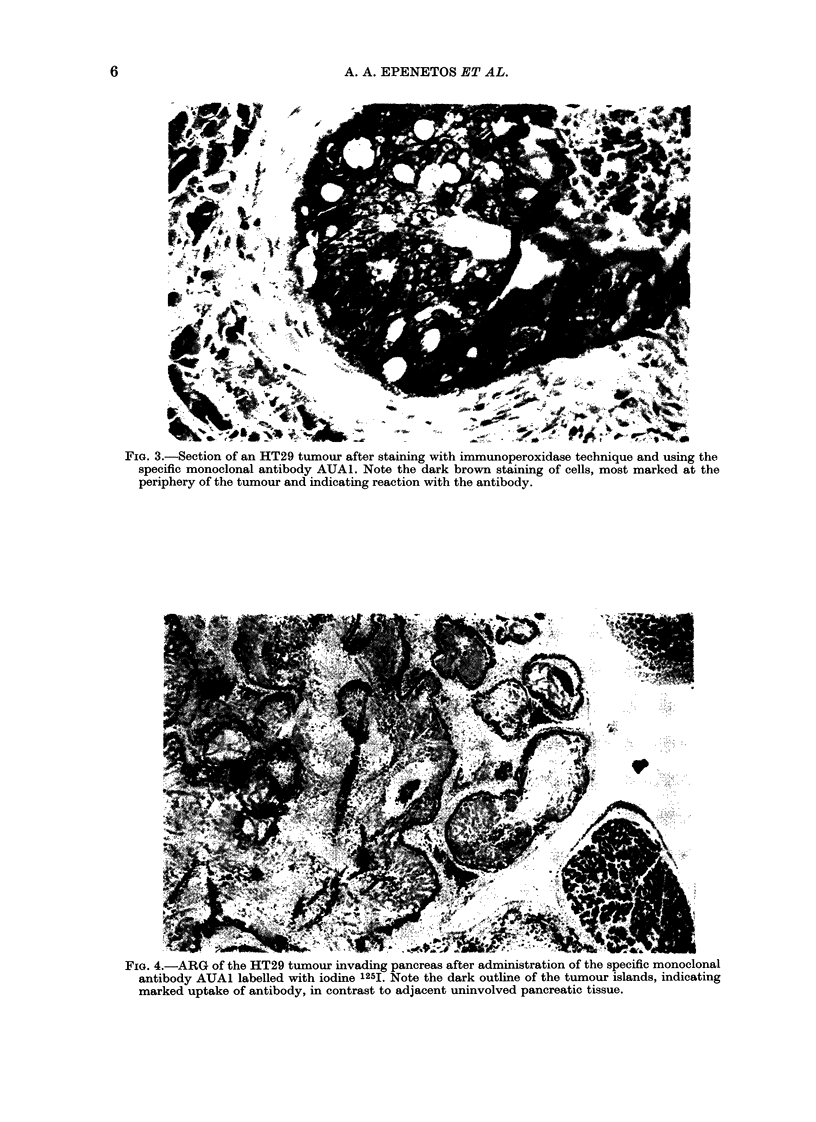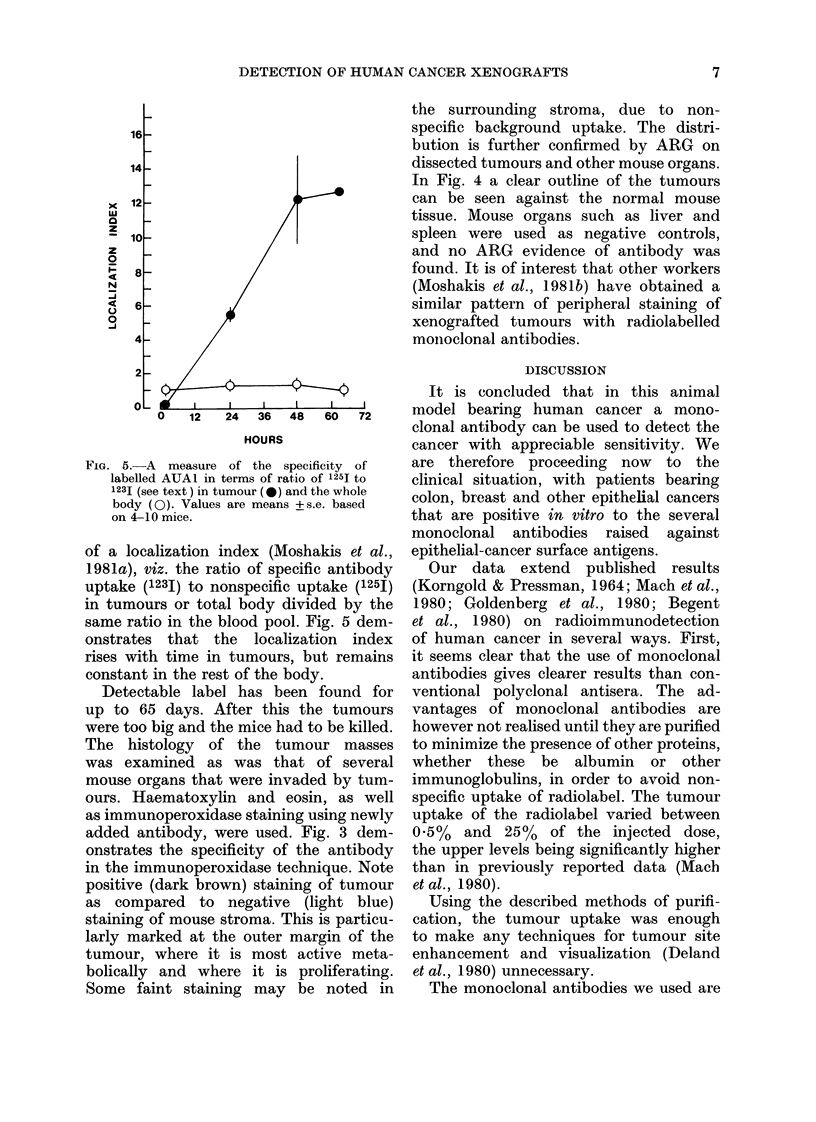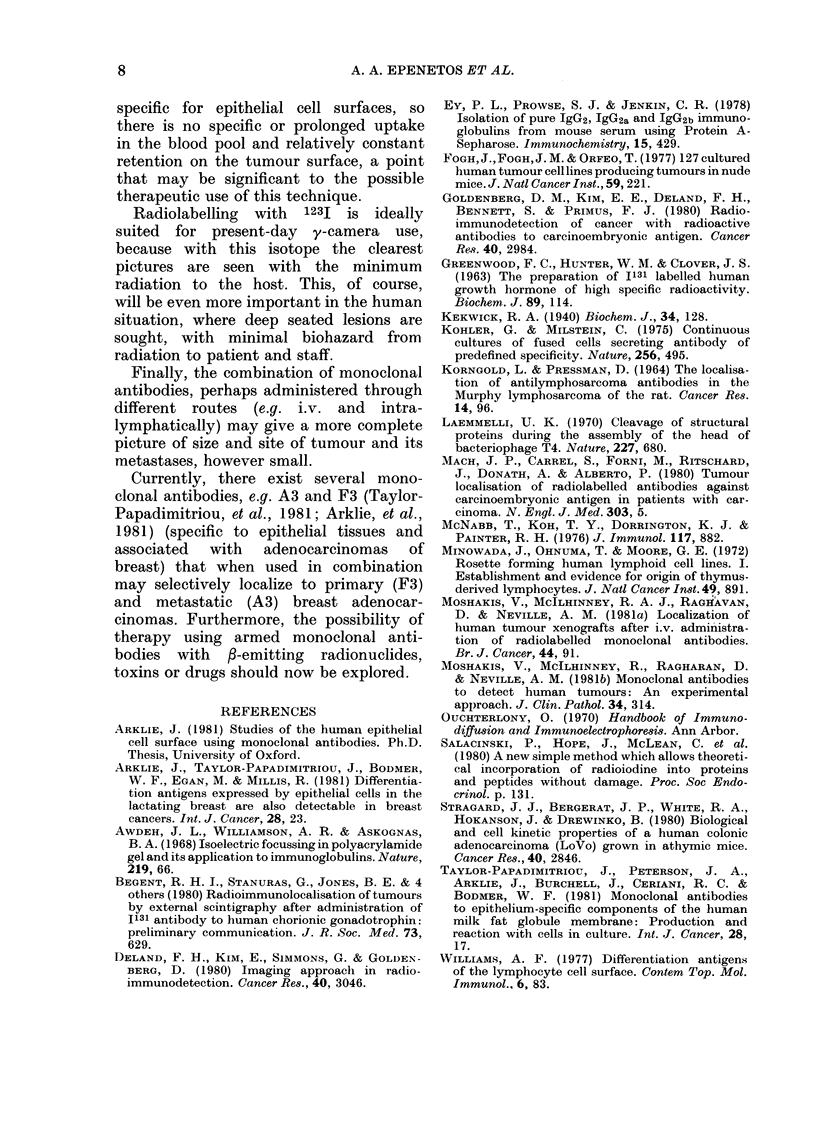# Detection of human cancer in an animal model using radio-labelled tumour-associated monoclonal antibodies.

**DOI:** 10.1038/bjc.1982.157

**Published:** 1982-07

**Authors:** A. A. Epenetos, C. C. Nimmon, J. Arklie, A. T. Elliott, L. A. Hawkins, R. W. Knowles, K. E. Britton, W. F. Bodmer

## Abstract

**Images:**


*Br. J. Cancer* (1982) **46**, 1

### DETECTION OF HUMAN CANCER IN AN ANIMAL MODEL USING RADIO-LABELLED TUMOUR-ASSOCIATED MONOCLONAL ANTIBODIES

A. A. EPENETOS*‡, C. C. NIMMON†, J. ARKLIE*, A. T. ELLIOTT†, L. A. HAWKINS†, R. W. KNOWLES*, K. E. BRITTON† AND W. F. BODMER*.

*From the *Imperial Cancer Research Fund, Lincoln's Inn Fields, London WC2A 3PX, the †Department of Nuclear Medicine, St Bartholomew's Hospital, London EC1 and the ‡Imperial Cancer Reserach Fund, Medical Oncology Unit, St Bartholomew's Hospital, London EC1*

Received 26 October 1981 Accepted 1 February 1982

**Summary.—Monoclonal antibodies to epithelial-cell antigenic determinants, labelled with $^{123}$I and $^{125}$I, were administered parenterally to immunodeficient mice bearing human tumours derived from a human cancer cell line. Anterior, posterior and lateral radioscans of the body were taken with a gamma scintillation camera at various times from immediately to 65 days after injection. Visual displays of the images were processed by standard computer techniques. The model used a human colon-cancer cell line, HT29, and the monoclonal antibody, AUA1, which is specific to an epithelial proliferating antigen. Tumour detection was achieved in all the mice. The smallest tumour detectable appeared to be about 1 mm in diameter. The degree of antibody uptake in a tumour depended on its size and the blood supply of its surrounding tissues We believe that the technology and skills are now available for accurate radio-immunodetection of cancer in man.**

THE LOCALIZATION OF CANCER in man using radionuclides has so far been non-specific, relating, for example, to a focal defect on a liver scan or a focal area of increased activity on a bone or a brain scan. The recognition of tumour-associated and highly tissue-specific antigens, the development by Kohler and Milstein (1975) of the monoclonal-antibody technique and the regular production by A.E.R.E. Harwell of pure iodine-123 bring together the requirements for accurate radioimmunodetection of cancer in man.

Radioimmunodetection of cancer is not a new field and its potential clinical use has been demonstrated by many authors in the past (Korngold & Pressman, 1964; Mach *et al.*, 1980; Goldenberg *et al.*, 1980; Begent *et al.*, 1980; Moshakis *et al.*, 1981*a*). This study, using a human cancer cell line in an animal model and a pure $^{123}$I-labelled monoclonal antibody, extends previous results.

### METHODS

### *Monoclonal antibodies*

The monoclonal antibody AUA1 was obtained from Arklie (1981), who raised it in the conventional fashion by immunizing BALB/c mice with the colon-carcinoma cell line LoVo (Stragard *et al.*, 1980). This antibody is specific for an epithelial proliferating antigen as found by testing in binding assays against epithelial-cancer cell lines and in sections using immunofluorescent and immunoperoxidase techniques and involving normal and malignant epithelial tissues (Arklie, 1981). Using immunoperoxidase staining, this antibody stains positively human colon carcinomas as well as the villi of normal colon. It is not a tumour-specific antibody, but it is tissue-specific and tumour-associated.

The negative-control monoclonal antibody M236 was obtained from Dr R. Knowles, who raised it against the lymphoblastoid cell line MOLT4. (Minowada *et al.*, 1972). This antibody has been found to be specific for cells of the T-lymphocyte lineage and does not react with cells of epithelial origin.

Both antibodies were grown in bulk, either as ascites in mice or as supernatants in tissue culture. The average concentration of antibodies was 2 mg/ml in ascites and 10 $\mu$g/ml in supernatant.

*Characterization of immunoglobulin*

AUA1 and M236 were found to belong to the $IgG_1$ subclass by Ouchterlony double diffusion. (Ouchterlony, 1970).

*Preparation of pure IgG.*—Ammonium sulphate precipitation (Kekwick, 1940) was used as a first step in separating the bulk of albumin from the immunoglobulin. The precipitate was then filtered through a protein-A–Sepharose column and the $IgG_1$ subfraction was eluted using a citrate buffer (pH 6·0) (Ey *et al.*, 1978). The purity of the $IgG_1$ was checked using polyacrylamide-gel electrophoresis (Laemmelli, 1970) and isoelectric focusing (Awdeh *et al.*, 1968). Both techniques showed a characteristic monoclonal pattern, indicating a highly purified product.

*Binding to cells.*—The reactivity of the fractions containing AUA1 and M236 was determined by binding to HT29 and MOLT4 cells in a radioimmune binding assay (Williams, 1977). Both antibodies were tested for reactivity, and were found to bind to their respective target cells after inodination.

*Iodination of monoclonal antibodies.*—The $IgG_1$ fractions were iodinated with $^{123}I$ or $^{125}I$ using 2 methods: Chloramine T (Greenwood *et al.*, 1963) and Iodogen (Salacinski *et al.*, 1980).

Both techniques were similar in efficacy, yielding about 80% incorporation of iodine into immunoglobulin when equimolar amounts of iodine and immunoglobulin were used in the initial reaction. The chloramine T method was as follows: 25 $\mu$l of antibody at 1 mg/ml in citrate buffer (pH 6·0) mixed with 25 $\mu$l of phosphate buffer (0·3M, pH 7·4) were reacted with 10 $\mu$l of $^{125}I$ (37 MBq, Amersham, 1 mC code 1 IMS 30 and 10 $\mu$l of chloramine T (2 mg/ml) in phosphate buffer (0·3M, pH 7·4). The reaction solution was mixed for 2 min. Free iodine was removed by gel filtration, using a G50 Sephadex column and eluting with phosphate-buffered saline (PBSA) The G50 Sephadex was prewashed with 1% BSA in PBSA. The iodogen method was as follows: 2 ml of iodogen (40 $\mu$g/ml) in dichloromethane was allowed to dry in small conical propylene tubes. Antibody, phosphate buffer and $^{125}I$ at the same amounts as used for the chloramine T method were added and left for 20 min. Again free iodine was removed using a G50 Sephadex column. In both techniques a specific activity of 5 mqB/$\mu$g was achieved.

When iodinating with $^{123}I$ (A.E.R.E., Harwell) the conditions were the same as for $^{125}I$, except that phosphate buffer (0·3M, pH 5·5) was used to adjust the final pH to 7·4, and 100 $\mu$l of $^{123}I$ in 0·04M NaoH (pH 12·4) equivalent to 37 MBq (1mC) were added. The iodination efficiency using $^{123}I$ was less then when using $^{125}I$, probably because $^{123}I$ is more dilute (it has a molarity of $4 \times 10^{-8}$M compared to $^{125}I$ of $5 \times 10^{-5}$M). The antibodies were injected into mice on the same day that they were iodinated.

The different iodine isotopes $^{123}I$ and $^{125}I$ were used for different purposes. $^{123}I$ gives maximum clarity of tumours at any depth of tissue. Although it has a half-life of only 13 h, adequate views were obtained up to 96 h after injection. The principal radiation of $^{123}I$ is a $\gamma$-ray with an energy of 160 keV, which is ideally suited for present-day $\gamma$-cameras.

$^{125}I$ is used to examine the long-term behaviour of the labelled monoclonal antibody in the tumour. Studies with $^{125}I$ have been carried out sequentially up to 65 days from injection of antibody. $^{125}I$ also offers the opportunity for autoradiographs on tumour masses and other organs.

The monoclonal antibody M236 labelled with $^{125}I$ and $^{123}I$ was used as a negative control. A monoclonal antibody of the same immunoglobulin subclass ($IgG_1$) provides the most appropriate comparison between specific and nonspecific uptake. Subtraction of the 2 results (*i.e.* the M236 from the AUA1) was not found to be necessary for visualization of the tumour.

*Immunoperoxidase staining*

Formalin-fixed and paraffin-embedded sections of tumours and other organs were first dewaxed and then fixed in alcohol. Sections were tested in triplicate: 1 with the monoclonal antibody of interest (AUA1), 1 with the negative-control antibody (M236)

and 1 without antibody to assess the nonspecific background staining (Arklie, 1981).

### *Autoradiography*

The selective localization of injected iodinated monoclonal antibody in tumours, in contradistinction to the absence from other normal organs, is demonstrated by autoradiography (ARG). Formalin-fixed and paraffin-embedded sections of tumours and other organs are first dewaxed and then fixed in alcohol. ARGs were prepared using Ilford K5 fluid emulsion. After drying in air the sections were exposed for 7–30 days at 4°C. The sections were then developed, fixed and stained with haemotoxylin for light microscopy.

### *Scanning*

Nineteen nude mice and 1 nude rat were tested. Before scanning, the animals were anaesthetized with i.p. pentobarbitone. The amount of injected radiolabel ranged from 0·08 mC (3 MBq) to 0·3 mC (11·0 MBq) for $^{123}$I, and from 0·1 mC (3·7 MBq) to 20 mC (740 MBq) of $^{125}$I. The amount of antibody administered ranged from 2 $\mu$g to 500 $\mu$g. (500 $\mu$g, equivalent to 740 MBq = 20 mC was given only once to a nude rat as a therapeutic attempt). All the mice were injected with antibody in the range 2–10 $\mu$g. Imaging was performed with a standard $\gamma$-camera fitted with a high-sensitivity collimator. The camera was linked to a computer with data display. The counts at different regions of interest (*i.e.* total body, tumour, blood pool) were then calculated and expressed as a percentage of the initial injected amount. For each selected region of interest, a narrow circumferential region was used as a background area to allow estimation of uptake. This technique enables us to quantitate the uptake of antibody at different regions of interest sequentially, and thus get information both on the uptake of the antibody and also on its catabolism and excretion without having to sacrifice the animal. If, for example, one counts the activity over a tumour region and the tumour is then removed and counted using standard scintillation counting, the same count is obtained on both occasions. Thus this technique, though different in offering an opportunity to study the dynamic behaviour of the radiolabelled antibody, does not give different counts from conventional scintillation counting.

### RESULTS

Tumour localization was achieved in all the animals. A typical radioscan is shown in Fig. 1(a), where a mouse with an i.m. HT29 tumour (Fogh *et al.*, 1977) on its left leg was scanned at 20 min and 2, 18 and 48 h after an i.v. injection of AUA1 monoclonal antibody labelled with $^{123}$I. As can be seen, after 18 h the tumour is clearly visible. Fig. 1(b) shows scans of the same mouse after being injected with the nonspecific antibody M236 labelled with $^{125}$I, also scanned at 20 min and 2, 18 and 48 h. The scans taken at 18 and 48 h show some activity over the tumour region, calculated to be 1% and 0·1% of the injected amount, respectively. This uptake is spurious, because 30% of the counts arising from AUA1 $^{123}$I are picked up in the $^{125}$I channel. However, to some extent there is nonspecific uptake of Ig, as demonstrated by other experiments in which tumour-bearing mice were injected with the nonspecific antibody alone. The nonspecific uptake of antibody will depend on at least 2 factors. Firstly, tumours may have Fc receptors and thus be able to capture some Ig if it is aggregated. Secondly, the vascularity of a tumour will influence its blood supply and also the nonspecific trapping of antibody. We have performed similar experiments in other tumour models, using a trophoblastic cell line (Dosmi) and neuroblastoma cell line (TR14) and found that both of these grow as vascular tumours and have much higher uptake of nonspecific antibodies. In the case of the trophoblastic cell line Dosmi we found no difference between nonspecific and specific antibody uptake. When Dosmi cells were grown as i.m. tumours, we obtain 30–40% uptake of the injected amount of *any* radiolabelled antibody. This is probably due to the special properties of the trophoblast for example, their large number of Fc-receptor sites (McNabb *et al.*, 1976). A further point, related to the first two, is of course the size of the tumour. Nonspecific uptake increases proportionately with the size

FIG. 1.—Radioscans of a mouse with an i.m. HT29 tumour on its left leg taken at 20 min, 2 h, 18 h and 48 h after injection of monoclonal labelled antibody (a) Specific AUA1 labelled with $^{123}I$. Note clearly visible tumour at 18 h and 48 h. (b) Nonspecific M236 labelled with $^{125}I$. Very little activity is seen over the tumour area and 30% of this is due to the counts, arising from AUA1 labelled with $^{123}I$ and picked up in the $^{125}I$ channel.

FIG. 2.—Graphical display of uptake by total body tumour in mice bearing i.m. and s.c. HT29 tumours. The circles indicate the uptake of the specific antibody and the triangles the uptake of the nonspecific antibody. Each point represents 5–11 mice. Error bars show s.e.

of the tumour. We tried to use relatively small tumours from just palpable to 1 cm diameter. Moreover the HT29 cells grow as rather avascular and centrally necrotic tumour masses and this explains the low uptake of the nonspecific M236 antibody with this model. Fig. 2 shows the uptake of both specific and non-specific antibodies. The highest and fastest specific antibody uptake was seen in the i.m. tumours, with uptake ranging from 0·5% to 25% of the injected dose (mean ~6·0%). This level was reached between 4 and 18 h after injection (see Fig. 2). The average uptake of the nonspecific antibody was ~0·15% of the injected dose.

Both the i.p. and s.c. tumours had an uptake of ~0·5%, but it is interesting to note that in the i.p. tumours the antibody localized faster (*i.e.* within 24 h) while in the s.c. tumours the uptake was slower, taking up to 6 days to reach maximum level. There was no measurable (*i.e.* <0·1%) uptake of nonspecific antibody in the s.c. tumours.

The best pictures are seen after a period ranging from 4 h to 1 week, depending again on the site of the tumour. For example, most of the i.m. tumours were clearly seen after 18 h, because the uptake in the tumour, after reaching its peak, remained relatively high for much longer than in the rest of the body, whence it was mostly cleared by 18 h, thus leaving a "hotter" spot in the tumour area. These results could also be expressed in terms

FIG. 3.—Section of an HT29 tumour after staining with immunoperoxidase technique and using the specific monoclonal antibody AUA1. Note the dark brown staining of cells, most marked at the periphery of the tumour and indicating reaction with the antibody.

FIG. 4.—ARG of the HT29 tumour invading pancreas after administration of the specific monoclonal antibody AUA1 labelled with iodine $^{125}I$. Note the dark outline of the tumour islands, indicating marked uptake of antibody, in contrast to adjacent uninvolved pancreatic tissue.

FIG. 5.—A measure of the specificity of labelled AUA1 in terms of ratio of $^{125}I$ to $^{123}I$ (see text) in tumour (●) and the whole body (○). Values are means ±s.e. based on 4–10 mice.

of a localization index (Moshakis *et al.*, 1981*a*), *viz.* the ratio of specific antibody uptake ($^{123}I$) to nonspecific uptake ($^{125}I$) in tumours or total body divided by the same ratio in the blood pool. Fig. 5 demonstrates that the localization index rises with time in tumours, but remains constant in the rest of the body.

Detectable label has been found for up to 65 days. After this the tumours were too big and the mice had to be killed. The histology of the tumour masses was examined as was that of several mouse organs that were invaded by tumours. Haematoxylin and eosin, as well as immunoperoxidase staining using newly added antibody, were used. Fig. 3 demonstrates the specificity of the antibody in the immunoperoxidase technique. Note positive (dark brown) staining of tumour as compared to negative (light blue) staining of mouse stroma. This is particularly marked at the outer margin of the tumour, where it is most active metabolically and where it is proliferating. Some faint staining may be noted in the surrounding stroma, due to nonspecific background uptake. The distribution is further confirmed by ARG on dissected tumours and other mouse organs. In Fig. 4 a clear outline of the tumours can be seen against the normal mouse tissue. Mouse organs such as liver and spleen were used as negative controls, and no ARG evidence of antibody was found. It is of interest that other workers (Moshakis *et al.*, 1981*b*) have obtained a similar pattern of peripheral staining of xenografted tumours with radiolabelled monoclonal antibodies.

### DISCUSSION

It is concluded that in this animal model bearing human cancer a monoclonal antibody can be used to detect the cancer with appreciable sensitivity. We are therefore proceeding now to the clinical situation, with patients bearing colon, breast and other epithelial cancers that are positive *in vitro* to the several monoclonal antibodies raised against epithelial-cancer surface antigens.

Our data extend published results (Korngold & Pressman, 1964; Mach *et al.*, 1980; Goldenberg *et al.*, 1980; Begent *et al.*, 1980) on radioimmunodetection of human cancer in several ways. First, it seems clear that the use of monoclonal antibodies gives clearer results than conventional polyclonal antisera. The advantages of monoclonal antibodies are however not realised until they are purified to minimize the presence of other proteins, whether these be albumin or other immunoglobulins, in order to avoid nonspecific uptake of radiolabel. The tumour uptake of the radiolabel varied between 0·5% and 25% of the injected dose, the upper levels being significantly higher than in previously reported data (Mach *et al.*, 1980).

Using the described methods of purification, the tumour uptake was enough to make any techniques for tumour site enhancement and visualization (Deland *et al.*, 1980) unnecessary.

The monoclonal antibodies we used are specific for epithelial cell surfaces, so there is no specific or prolonged uptake in the blood pool and relatively constant retention on the tumour surface, a point that may be significant to the possible therapeutic use of this technique.

Radiolabelling with $^{123}I$ is ideally suited for present-day $\gamma$-camera use, because with this isotope the clearest pictures are seen with the minimum radiation to the host. This, of course, will be even more important in the human situation, where deep seated lesions are sought, with minimal biohazard from radiation to patient and staff.

Finally, the combination of monoclonal antibodies, perhaps administered through different routes (*e.g.* i.v. and intra-lymphatically) may give a more complete picture of size and site of tumour and its metastases, however small.

Currently, there exist several monoclonal antibodies, *e.g.* A3 and F3 (Taylor-Papadimitriou, *et al.*, 1981; Arklie, *et al.*, 1981) (specific to epithelial tissues and associated with adenocarcinomas of breast) that when used in combination may selectively localize to primary (F3) and metastatic (A3) breast adenocarcinomas. Furthermore, the possibility of therapy using armed monoclonal antibodies with $\beta$-emitting radionuclides, toxins or drugs should now be explored.

### REFERENCES

ARKLIE, J. (1981) Studies of the human epithelial cell surface using monoclonal antibodies. Ph.D. Thesis, University of Oxford.

ARKLIE, J., TAYLOR-PAPADIMITRIOU, J., BODMER, W. F., EGAN, M. & MILLIS, R. (1981) Differentiation antigens expressed by epithelial cells in the lactating breast are also detectable in breast cancers. *Int. J. Cancer*, **28**, 23.

AWDEH, J. L., WILLIAMSON, A. R. & ASKOGNAS, B. A. (1968) Isoelectric focussing in polyacrylamide gel and its application to immunoglobulins. *Nature*, **219**, 66.

BEGENT, R. H. I., STANURAS, G., JONES, B. E. & 4 others (1980) Radioimmunolocalisation of tumours by external scintigraphy after administration of $I^{131}$ antibody to human chorionic gonadotrophin: preliminary communication. *J. R. Soc. Med.* **73**, 629.

DELAND, F. H., KIM, E., SIMMONS, G. & GOLDENBERG, D. (1980) Imaging approach in radioimmunodetection. *Cancer Res.*, **40**, 3046.

EY, P. L., PROWSE, S. J. & JENKIN, C. R. (1978) Isolation of pure $IgG_2$, $IgG_{2a}$ and $IgG_{2b}$ immunoglobulins from mouse serum using Protein A-Sepharose. *Immunochemistry*, **15**, 429.

FOGH, J., FOGH, J. M. & ORFEO, T. (1977) 127 cultured human tumour cell lines producing tumours in nude mice. *J. Natl Cancer Inst.*, **59**, 221.

GOLDENBERG, D. M., KIM, E. E., DELAND, F. H., BENNETT, S. & PRIMUS, F. J. (1980) Radioimmunodetection of cancer with radioactive antibodies to carcinoembryonic antigen. *Cancer Res.* **40**, 2984.

GREENWOOD, F. C., HUNTER, W. M. & CLOVER, J. S. (1963) The preparation of $I^{131}$ labelled human growth hormone of high specific radioactivity. *Biochem. J.* **89**, 114.

KEKWICK, R. A. (1940) *Biochem. J.*, **34**, 128.

KOHLER, G. & MILSTEIN, C. (1975) Continuous cultures of fused cells secreting antibody of predefined specificity. *Nature*, **256**, 495.

KORNGOLD, L. & PRESSMAN, D. (1964) The localisation of antilymphosarcoma antibodies in the Murphy lymphosarcoma of the rat. *Cancer Res.* **14**, 96.

LAEMMELLI, U. K. (1970) Cleavage of structural proteins during the assembly of the head of bacteriophage T4. *Nature*, **227**, 680.

MACH, J. P., CARREL, S., FORNI, M., RITSCHARD, J., DONATH, A. & ALBERTO, P. (1980) Tumour localisation of radiolabelled antibodies against carcinoembryonic antigen in patients with carcinoma. *N. Engl. J. Med.* **303**, 5.

MCNABB, T., KOH, T. Y., DORRINGTON, K. J. & PAINTER, R. H. (1976) *J. Immunol.* **117**, 882.

MINOWADA, J., OHNUMA, T. & MOORE, G. E. (1972) Rosette forming human lymphoid cell lines. I. Establishment and evidence for origin of thymus-derived lymphocytes. *J. Natl Cancer Inst.* **49**, 891.

MOSHAKIS, V., MCILHINNEY, R. A. J., RAGHAVAN, D. & NEVILLE, A. M. (1981*a*) Localization of human tumour xenografts after i.v. administration of radiolabelled monoclonal antibodies. *Br. J. Cancer*, **44**, 91.

MOSHAKIS, V., MCILHINNEY, R., RAGHARAN, D. & NEVILLE, A. M. (1981*b*) Monoclonal antibodies to detect human tumours: An experimental approach. *J. Clin. Pathol.* **34**, 314.

OUCHTERLONY, O. (1970) *Handbook of Immunodiffusion and Immunoelectrophoresis.* Ann Arbor.

SALACINSKI, P., HOPE, J., MCLEAN, C. *et al.* (1980) A new simple method which allows theoretical incorporation of radioiodine into proteins and peptides without damage. *Proc. Soc Endocrinol.* p. 131.

STRAGARD, J. J., BERGERAT, J. P., WHITE, R. A., HOKANSON, J. & DREWINKO, B. (1980) Biological and cell kinetic properties of a human colonic adenocarcinoma (LoVo) grown in athymic mice. *Cancer Res.*, **40**, 2846.

TAYLOR-PAPADIMITRIOU, J., PETERSON, J. A., ARKLIE, J., BURCHELL, J., CERIANI, R. C. & BODMER, W. F. (1981) Monoclonal antibodies to epithelium-specific components of the human milk fat globule membrane: Production and reaction with cells in culture. *Int. J. Cancer*, **28**, 17.

WILLIAMS, A. F. (1977) Differentiation antigens of the lymphocyte cell surface. *Contem Top. Mol. Immunol.*, **6**, 83.